# *Halomonas* sp. OKOH—A Marine Bacterium Isolated from the Bottom Sediment of Algoa Bay—Produces a Polysaccharide Bioflocculant: Partial Characterization and Biochemical Analysis of Its Properties

**DOI:** 10.3390/molecules16064358

**Published:** 2011-05-25

**Authors:** Leonard V. Mabinya, Sekelwa Cosa, Noxolo Mkwetshana, Anthony I. Okoh

**Affiliations:** Applied and Environmental Microbiology Research Group (AEMREG), Department of Biochemistry and Microbiology, University of Fort Hare, Private Bag X1314, Alice 5700, South Africa; Email: lmabinya@ufh.ac.za (L.V.M.); sekco@webmail.co.za (S.C.); nmkwetshana@ufh.ac.za (N.M.)

**Keywords:** *Halomonas* sp. OKOH, bioflocculant, polysaccharide

## Abstract

A bioflocculant-producing bacterium isolated from seawater was identified based on 16S rRNA gene nucleotide sequence to have 99% similarity to that of *Halomonas* sp. Au160H and the nucleotide sequence was deposited as *Halomonas* sp. OKOH (Genbank accession number is HQ875722). Influences of carbon source, nitrogen source, salt ions and pH on flocculating activity were investigated. The bioflocculant was optimally produced when glucose (87% flocculating activity) and urea (88% flocculating activity) were used as sources of carbon and nitrogen, respectively. Also, initial pH of 7.0 and Ca^2+^ supported optimal production of the bioflocculant with flocculating activities of 87% respectively. Chemical analyses revealed the bioflocculant to be a polysaccharide.

## 1. Introduction

Flocculants are used for the aggregation of colloidal substances and cellular materials and thus are widely applied in different industrial processes, including wastewater treatment, downstream processing, food and fermentation processes [1,2]. Inorganic flocculating agents, such as aluminum sulfate, polyaluminum chloride, ferric chloride and organic polymers such as polyacrylamide derivatives, are frequently used in both wastewater treatment and the fermentation industries because they are not only cost-effective, but also have strong flocculating activity [3]. However, studies have shown that some of the chemically synthetic flocculating substances are not only harmful to both humans and the environment, but are also non-degradable in Nature [4,5]. 

A bioflocculant on the other hand, is a kind of biodegradable polymeric flocculant produced by many microorganisms including bacteria, fungi and actinomycetes during their growth [3,6]. Compared with conventional synthetic organic flocculants, bioflocculants have special advantages such as safety for ecosystems, potential flocculating effects, biodegradability and harmlessness to humans and the environment, and as a consequence may potentially be applied in drinking and wastewater treatment, downstream processing, food and fermentation processes [1,2]. The interest in biotechnological methods for the production of bioflocculants lies in the possibility of using different organisms to synthesize extracellular substances with different compositions [4,7,8].

Many microorganisms, including bacteria, fungi and actinomycetes, have been reported to produce extracellular polymeric substances, such as polysaccharides, functional proteins and glycoproteins, which function as bioflocculants [3,6]. Studies carried out on the chemical composition of bioflocculants produced by *Bacillus* sp. I-471 [11], *Halomonas* sp. V3׳a [13,14], *Bacillus subtilis* DYU1 [12], *Vagococcus* sp. W31 [6], and *Bacillus subtilis* IFO3335 [15] have shown them to all be polysaccharides. *Rodoccocus erythropolis* [9] and *Nocardia amarae* YK-1 [10] produce protein flocculants while *Arcuadendron* sp. TS-4 [16] and *Arathrobacter* sp. [17] have been reported to produce glycoprotein bioflocculants.

In spite of all the bioflocculants that have been identified, few of them have been applied practically in industry because some require further enhancement of flocculating capability in order to make dosage requirements cost-effective [6]. Hence, there is need to identify new microorganisms (especially from unusual environments like the marine habitat) with high bioflocculant-producing ability and/or improve upon the flocculating efficiency of the known bioflocculants. 

The presence of indigenous marine microorganisms in the oceans have been confirmed by recent studies which also indicate that they are widely distributed in different marine habitats, from ocean shores [18] to the deep sea floor and coral reefs [19]. Since marine microorganisms have evolved the greatest genomic and metabolic diversity, efforts should be directed towards exploring them as a potential source for the discovery of novel secondary metabolites [20]. 

As marine environmental conditions are extremely different from terrestrial ones, it is surmised that marine microorganisms have different characteristics from those of terrestrial counterparts and, therefore, might produce different types of bioactive compounds. The living conditions to which marine microorganisms had to adapt during evolution range from extremely high pressure (with a maximum of about 1100 atmospheres) and anaerobic conditions at temperatures just below 0 °C on the deep sea floor, to high acidic conditions (pH as low as 2.8) at temperatures of over 100 °C near hydrothermal vents at the mid-ocean ridges [20]. It is therefore likely that this is reflected in the genetic and metabolic diversity of these microorganisms which remains largely unknown [21,22,23,24]. 

The genus *Halomonas* is comprised of moderately halophilic, Gram-negative rods whose species are widely distributed in hypersaline habitats [25]. In this paper we report of the bioflocculant production potential of *Halomonas* sp. OKOH isolated from the bottom sediment of Algoa Bay in the Eastern Cape Province of South Africa.

## 2. Results and Discussion

### 2.1. Identification of the Test Bacterium

**Figure 1:**
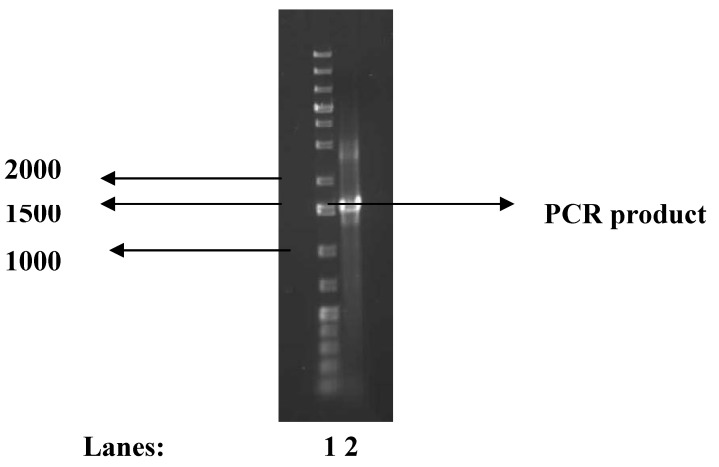
Agarose gel electrophoresis of PCR product of *Halomonas* sp. OKOH. Lanes: 1 = Ladders, 2 = PCR product.

The test bacterium was amongst the several bacteria isolated from the bottom sediments of Algoa Bay in the Eastern Cape Province of South Africa. This bioflocculant-producing bacterium grew well in the presence of NaCl (seawater) indicating that it was halotolerant and also exhibited optimum growth at pH 7. The identity of the bioflocculant producing-bacterium was confirmed by 16S rRNA gene nucleotide sequence analysis following PCR amplification of the gene. The amplified fragments were analyzed by electrophoresis on a 1% agarose gel stained with ethidium bromide using Mass Ruler DNA Ladders and a PCR product with the size of approximately 1.5 kb was yielded (Figure 1). 16S rRNA gene sequencing was performed using Spectrumedix SCE2410 genetic analysis system which revealed a 99% similarity of the test bacterium to *Halomonas* sp. Au160H. The bacterial strain was named *Halomonas* sp. OKOH and the sequence was therefore deposited in the Genbank with accession number HQ875722. A 16S rRNA gene sequence similarity higher than 97% is an indication that the new strain belongs to the same species [26,27,28].

The genus *Halomonas* is comprised of Gram-negative rods which are extremophiles, varying from moderate halophiles such as *Halomonas* *elongate*, to extreme halophiles such as *Halobacterium* *salinarum*. In order to survive and adapt in high ionic strength environments such as the sea, halophiles produce proteins which are designed to function under these conditions and in addition, are also capable of growing anaerobically in the presence of glucose [29].

### 2.2. Time Course Assay of Bioflocculant Production

In this study, the bioflocculant produced by *Halomonas* sp. OKOH growing on filtered seawater medium supplemented with different carbon and nitrogen sources was evaluated for its bioflocculant properties. According to Sam *et al*. [30], the flocculating capability of flocculants produced by halophilic bacterial strains is enhanced when the evaluation is carried out in seawater suspensions. *Halomonas maura* sp. nov. was also reported to have a strict growth requirement for sea salts with no growth observed in their absence [25]. In addition, the high-salinity condition of seawater tends to eliminate any risk of contamination during microbial cultivation [30].

**Figure 2:**
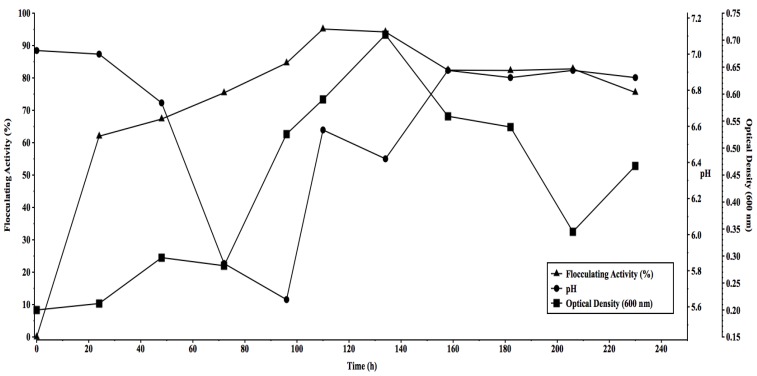
Time course assay of flocculant production by by *Halomonas* sp. OKOH. Legend are as follows: ■ (pH); ▲ ([flocculating activity (%)]; ● [optical density (600 nm)].

The growth pattern, flocculating activity and pH variation of the culture broth were as shown in Figure 2. During growth, the flocculating activity increased as the cultivation period increased, attaining a peak flocculating activity of about 95% after 135 h of cultivation, beyond which flocculating activity began to decline (Figure 2). The observed decrease in flocculating activity might be due to partial enzymatic degradation of the polymer flocculant in the late phases of cell growth [31]. He *et al.* [3] reported a decrease in flocculating activity of a bioflocculant produced by *Corynebacterium glutamicum* which was attributed to a decrease in the molecular weight of the polymer when it was subjected to protease hydrolysis. A corresponding increase in optical density with cultivation time was also observed and ran almost parallel to the flocculating activity curve (Figure 2). The observed direct relationship between growth and activity is an indication that the bioflocculant was produced by biosynthesis during growth of the bacterium and not by cell autolysis [3,6]. Figure 2 also shows a decrease in the pH of the culture broth from about 7.0 to about 5.6 during the first 96 h of incubation. This observed decrease in pH may, according to Dermlin *et al*. [32], be caused by the production of organic acids from either the metabolism of glucose, or from the produced bioflocculant, although a contrary observation was reported for *Halomonas maura* sp. nov. [25] where no such acid production was observed. The observed increase in pH in the later stages of cultivation could not be accounted for, but may be attributed to excretion of extracellular materials resulting from the partial degradation of the bioflocculant.

### 2.3. Chemical Analysis of Bioflocculant Composition

Total carbohydrate was determined by the phenol-sulphuric acid method, which gave a total carbohydrate content of 23 mg/mL, thus confirming the presence of carbohydrates as a major component of the bioflocculant produced by the bacterium. No obvious protein absorption peak was detected at 280 nm and further quantification of protein content using the Folin Lowry method proved negative. These findings corroborated the results obtained from chemical analyses of bioflocculants produced by other halophilic species belonging to the same genus, such as *Halomonas maura* sp. Nov [25], *Halomonas* sp. V3a’ [13,14], and *Halomonas* sp. AAD6 [30] which indicated the presence of polysaccharide as the main component. In their investigation of the chemical nature of the polymer flocculant produced by a haloalkalophilic *Bacillus* sp. I-450, Kumar *et al*. [11] also showed it to be composed of a polysaccharide. According to Sam *et al*. [30], the low charge density of polymers such as polysaccharides usually leads to enhanced performance in suspensions with high ionic strengths. Other microorganisms producing different kinds of polysaccharide flocculants have been investigated. These include *Anabaena* sp. [31], *Klebsiella pneuminiae* [1], *Corynebacterium glutamicum* [3] and *Vagococcus* sp. W31 [6].

### 2.4. Factors Affecting Bioflocculant Production

**Figure 3:**
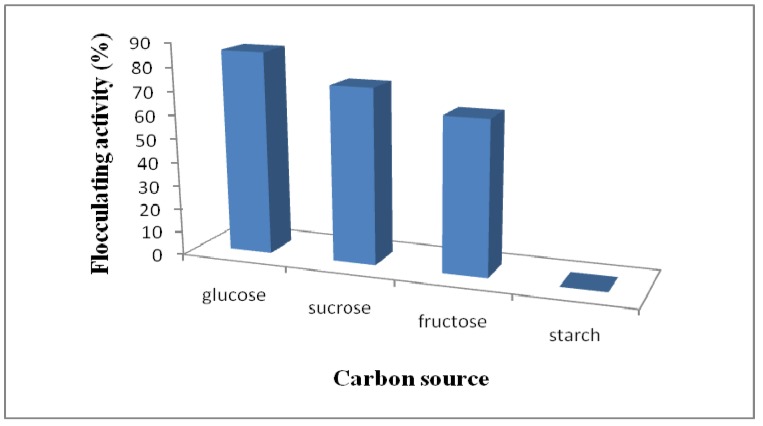
Effect of carbon source on flocculating activity of *Halomonas* sp. OKOH broth.

It has been well documented that changing the carbon and nitrogen sources highly influences bacterial growth and bioflocculant production [33]. Different carbon and nitrogen sources were also evaluated for their effects on flocculant production by this bacterium. From Figure 3, glucose seems to be the preferred carbon source for bioflocculant production by the bacterium, as it yielded about 87% flocculating activity compared to sucrose, fructose and starch, which yielded about 75%, 66% and 0% flocculating activities, respectively. 

Glucose was also reported as the carbon source of choice for enhancing the production of bioflocculants by *Vagococcus* sp. W31 [6] and *Halomonas* sp. V3a’ [13]. However, in their study of the bioflocculant production by *Klebsiella pneuminiae*, Nakata and Kurane [1] found that the use of ethanol as a carbon source supported the highest production of the polysaccharide flocculant, while Kumar *et al*. [11] used an optimized cultivation medium containing corn starch in their investigation of bioflocculant production by *Bacillus* sp. I-450.

**Figure 4:**
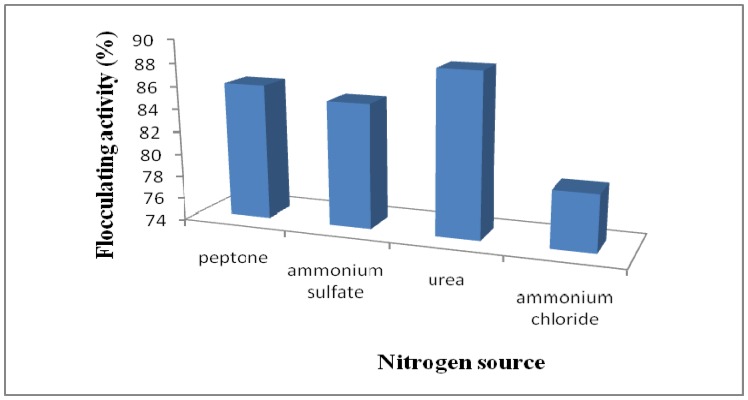
Effect of nitrogen source on flocculating activity of *Halomonas* sp. OKOH broth.

With respect to nitrogen source, urea gave the optimal production of bioflocculant with the highest flocculating activity of about 88.4%, compared to peptone, ammonium sulphate and ammonium chloride (Figure 4). Urea was also the preferred nitrogen source for the cultivation of haloalkalophilic *Bacillus* sp. I-450 [11] and *Vagococcus* sp. W31 [6]. This observation is contrary to the findings of He *et al*. [13] and Sam *et al*. [30] in which NH_4_Cl and peptone were found to be significant factors affecting bioflocculant production by *Halomonas* sp*.* V3a’ and *Halomonas* sp. AAD6, respectively. In addition, Bouchotroch *et al*. [25] reported a requirement for an L-amino acid such as alanine, arginine, histidine or leucine as the sole nitrogen source for the growth of *Halomonas maura* sp. nov.

### 2.5. Factors Affecting Bioflocculant Activity

**Figure 5:**
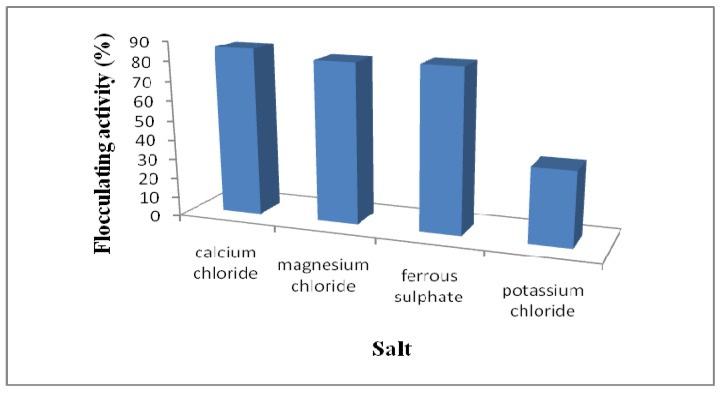
Effect of cations on bioflocculant activity by *Halomonas* sp. OKOH.

A dosage of the bioflocculant solution was mixed with kaolin solution supplemented with commercial cationic electrolytes and the rate of flocculation measured spectrophotometrically at 550 nm. Solutions of CaCl_2_, MgCl_2_, FeSO_4_·7H_2_O and KCl were used as sources of cations. As shown in Figure 5, flocculating activity was markedly increased by the addition of divalent cations with Ca^2+^ being marginally better than Mg^2+^ and Fe^2+^ in this respect. CaCl_2_ was also reported to significantly increase the flocculating activity of the bioflocculants MBFW31 produced by *Vagococcus* sp. W31 [6] and HBF-3 produced by a mutant *Halomonas* sp. V3a’ [14]. For *Serratia ficaria*-produced bioflocculant, both divalent cations, Ca^2+^ and Mg^2+^ enhanced the flocculating activity whereas the co-presence of trivalent cations Al^3+^ and Fe^3+^ negatively affected the flocculating activity [8]. On the other hand, Li *et al*. [34] noted an increase in flocculating activity in the presence of monovalent cations K^+^ and Na^+^ and a divalent cation Ca^2+^ for a bioflocculant produced by *Aeromonas* sp. Cations have been reported to be capable of neutralizing negatively charged functional groups of both the bioflocculant molecules and the suspended particles [2,35] and consequently weaken the static repulsive force thus enhancing the flocculation effect [7,11,35].

**Figure 6:**
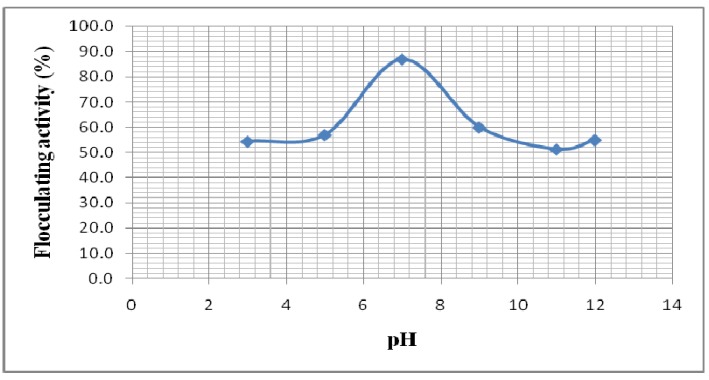
Effect of pH on flocculating activity.

The effect of initial pH of the culture medium on flocculating activity was examined at pH values ranging from 3-12 (Figure 6). The activity was found to be highest (87%) at pH 7. Bouchtroch *et al*. [25] and Gao *et al*. [6] reported similar optimal pH values (pHs 7.2 and 7.0) for the activity of the bioflocculants ERSS-31 produced by *Halomonas* *maura* sp. nov. and MBFW31 produced by *Vagococcus* sp. W31, respectively. Bioflocculant HBF-3, produced by a mutant *Halomonas* sp. V3a’ also attained the highest flocculating activity at pH 7 [14]. At low pH, the absorption of H^+^ ions tends to weaken the bioflocculant-kaolin complex formation process and a similar effect is also observed at high pH values [14]. According to Li *et al*. [35], the mediating effect of Ca^2+^ appears to be strongest at neutral pH values. However, these observations differ from the results of studies carried out by Choi *et al*. [31] and Zheng *et al*. [7] in which the maximum flocculating activities of bioflocculants produced by *Anabaena* sp. and *Bacillus* sp. F19, respectively, were observed at pH 2. The bioflocculant produced by *Bacillus* sp. PY-90 was found to be actively high in an acidic pH range of 3.0 to 5.0 [36], whilst *Serratia ficaria* produced a bioflocculant effective over a weakly acidic pH range of 5.0 to 7.0 [8].

## 3. Experimental

### 3.1. The Test Bacterium

The test bacterium was amongst several isolated from the bottom sediments of Algoa Bay in the Eastern Cape Province of South Africa as part of the culture collections of the Applied and Environmental Microbiology Research Group (AEMREG), University of Fort Hare, Alice, South Africa. The bacterium was preserved on agar slants and the glycerol (20%) stocks maintained at −80 °C.

### 3.2. Identification of the Bioflocculant-Producing Microorganism

*DNA extraction*: DNA extraction was conducted *via* the boiling method whereby 2-3 colonies were suspended in sterile double distilled water (70 µL), were heated in a water bath at 100 °C for 10 min, allowed to cool for 5 min and thereafter centrifuged at 3000 rpm for 5 min. The supernatant was transferred to a clean tube and stored at 4 °C. This served as the template in the PCR assay. 

*PCR Amplification*: PCR was carried out in 50 µL reaction volume containing 2 mM MgCl_2_, 2 U Supertherm Taq polymerase, 150 mM of each dNTP, 0.5 mM of each primer (F1: 59-AGAGTTTGATCITGGCTCAG-39; I = inosine and primer R5: 59-ACGGITACCTTGTTACGAC TT-39) and template DNA (2 mL). Primer F1 and R5 binds to base positions 7-26 and 1496-1476 of the 16S rRNA gene of *Streptomyces ambofaciens* ATCC 23877, respectively [37]. The primers in this study were used to amplify nearly full-length 16S rDNA sequences. The PCR programme used was an initial denaturation (96 °C for 2 min), 30 cycles of denaturation (96 °C for 45 s), annealing (56 °C for 30 s) and extension (72 °C for 2 min), and a ﬁnal extension (72 °C for 5 min). Gel electrophoresis of PCR products were conducted on 1% agarose gels to confirm that a fragment of the correct size had been ampliﬁed. 

Automated sequencing of the 16S rRNA genes of the bacterial isolates was performed using the Spectrumedix SCE2410 genetic analysis system with 24 capillaries. The sequencing reactions were performed according to the manufacturer’s instructions, using the Big Dye version 3.1 dye terminator cycle sequencing kit (Applied Biosystems) and 27F primer. The sequences were edited manually based on the most similar sequences.

### 3.3. Growth Medium and Cultivation Conditions

The composition of the growth medium was prepared by mixing the following: glucose (10 g), MgSO_4_∙7H_2_O (0.3 g), K_2_HPO_4_ (5 g), peptone (1.0 g) and KH_2_PO_4_ (0.2 g) in a litre of filtered seawater at pH 7.0 [38,39]. A loopful of colonies of the test bacterium was inoculated into the growth medium (5 mL) and incubated in a rotary shaker (160 rpm) at 28 °C for 5 days. The resultant culture broth (2 mL) was centrifuged (4000 × g, 30 min) and the cell-free supernatant was assayed for flocculating activity.

### 3.4. Time Course Assay of Bioflocculant Production

The composition of the medium for bioflocculant production was prepared according to the method described elsewhere [38,39]. The bacterium inoculum was prepared as a suspension in 0.9% saline solution and standardized to OD_660nm_ 0.1. This suspension was used to inoculate sterile culture broth (50 mL) in a 250 mL Erlenmeyer flask and incubated at 28 °C and 160 rpm for 16 hr for use as seed culture for batch fermentation. Batch fermentations were carried out according to the modified method of Gao *et al*. [6]. Seed culture (10% v/v) was used to inoculate medium (150 mL) in 500 mL flasks on a rotary shaker (160 rpm) at 28 °C. Culture samples were withdrawn at appropriate time intervals (24 hr) for a period of 240 hr and monitored for growth (OD_660_), pH and flocculating activity. Culture broth (2 mL) was centrifuged (4000 × g, 30 min.) and the cell-free supernatant was used to determine the flocculating activity as described in the next session.

### 3.5. Determination of Flocculating Activity

Using a suspension of kaolin clay as test material, flocculating activity was determined according to Kurane *et al*. [40] as modified by Gao *et al*. [6]. Kaolin clay was suspended in distilled water at a concentration of 5 g/L at pH 7 and used as a stock solution for the subsequent assays. The following solutions were mixed in a test tube: kaolin clay suspension (9 mL), culture supernatant (0.1 mL) and 1% CaCl_2_ (0.25 mL). A reference tube in which the culture supernatant was replaced with distilled water was also included and measured under the same conditions. The final volume of all mixtures was made up to 10 mL with distilled water. After mixing gently, the solutions were allowed to settle for 5 min. at room temperature. The optical density (OD) of the clarifying upper phase solution was measured at 550 nm with a UV spectrophotometer and the flocculating activity determined as follows:

[(B − A) / B] × 100%

where A and B are optical densities at 550 nm of the sample and control respectively.

### 3.6. Extraction, Purification and Characterization of the Bioflocculant

After 5 days of fermentation, the culture broth was centrifuged (8000 × g, 30 min.) to remove bacterial cells. One volume of distilled water was added to the supernatant phase and centrifuged again (8000 × g, 15 min.) to remove insoluble substances. The supernatant was then mixed with two volumes of ethanol, stirred and left standing at 4 °C for 12 h. The supernatant was decanted and the precipitate vacuum-dried to obtain the crude biopolymer. The crude product was then dissolved in distilled water and mixed with 1 volume of chloroform/*n*-butyl alcohol (5:2, v/v). After stirring, the mixture was left standing at room temperature for 12 h. The upper phase was separated, centrifuged (3000 × g, 15 min.) and the supernatant concentrated at 40 °C. Two volumes of ethanol were added, the precipitate recovered, vacuum-dried and re-dissolved in distilled water. Thereafter, the protein content was measured using the Folin-Lowry method, and carbohydrate was assayed using phenol-sulphuric acid protocol as described by Lachhwani [40].

### 3.7. Effect of Carbon and Nitrogen Sources on Bioflocculant Production

The effects of different carbon and nitrogen sources on bioflocculant production by the test bacterium were assessed. Carbon source candidates included glucose, sucrose, fructose and starch, while the nitrogen source candidates included ammonium sulphate, ammonium chloride (inorganic nitrogen sources) and urea (organic nitrogen sources) replacing peptone. The assessments were done in accordance with the description of Lachhwani [41]. 

### 3.8. Effect of Various Cations and pH on Bioflocculant Activity

Studying the effects of metal ions, flocculant tests was conducted utilizing the procedure elaborated above, but the CaCl_2_ solution was replaced by various metal salt solutions, and the flocculating activity was measured. Solutions of KCl, MgCl_2_ and FeSO_4_ were used as salt sources. To assess the effect of pH on flocculating activity, the pH of the reaction mixture was adjusted using HCl (0.1 M) and NaOH (0.1 M) in the pH range of 3-12 [42].

## 4. Conclusions

The bioflocculant produced by *Halomonas* sp. OKOH showed good flocculating activity for kaolin suspension. Glucose, urea and CaCl_2_ seem to be significant factors affecting the bioflocculant production by this microorganism. The addition of divalent cations enhances the flocculating activity at pH 7. Chemical analyses showed the novel bioflocculant to be a carbohydrate, a characteristic that gives it great potential for utilization not only in drinking and wastewater treatment processes but also in other processes such as food and fermentation industries.
